# Supplementary material for: The RNA binding proteins ZFP36L1 and ZFP36L2 are dysregulated in airway epithelium in human and a murine model of asthma
Source: Front Cell Dev Biol. 2023 Oct 19;11:1241008. doi: 10.3389/fcell.2023.1241008 (PMC10624177; doi:10.3389/fcell.2023.1241008)
Supplement: Supplementary file 1 [file DataSheet1.pdf]

Supplementary Material

The RNA binding proteins ZFP36L1 and ZFP36L2 are dysregulated in airway epithelium in human and a murine model of asthma

Jennifer Rynne, Elena Ortiz-Zapater, Dustin C. Bagley, Onofrio Zanin, George Doherty, Varsha Kanabar, David J. Jackson, Maddy Parsons, Jody Rosenblatt, Ian M. Adcock, Rocio T Martinez-Nunez*

*** Correspondence:** Rocio T. Martinez-Nunez: rocio.martinez_nunez@kcl.ac.uk

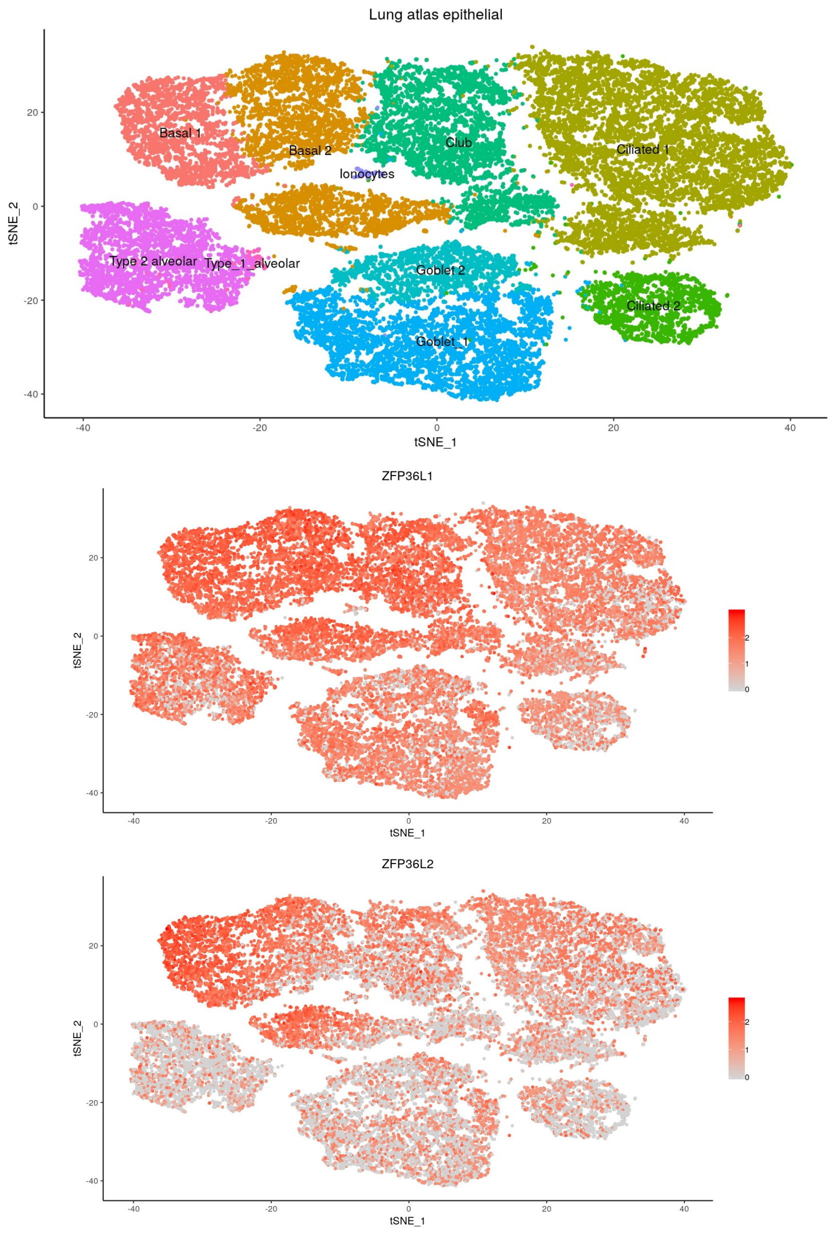

Supplementary Figure 1. Epithelial cell expression of *ZFP36L1* and *ZFP36L2* encoding transcripts. We interrogated the Lung Cell Atlas (https://asthma.cellgeni.sanger.ac.uk/) to determine the main airway epithelial cell type that presents *ZFP36L1* and *ZFP36L2* mRNA expression. While *ZFP36L1* was more diffusely present, *ZFP36L2* expression was more restricted to Basal 1 and Basal 2 type epithelial cells.

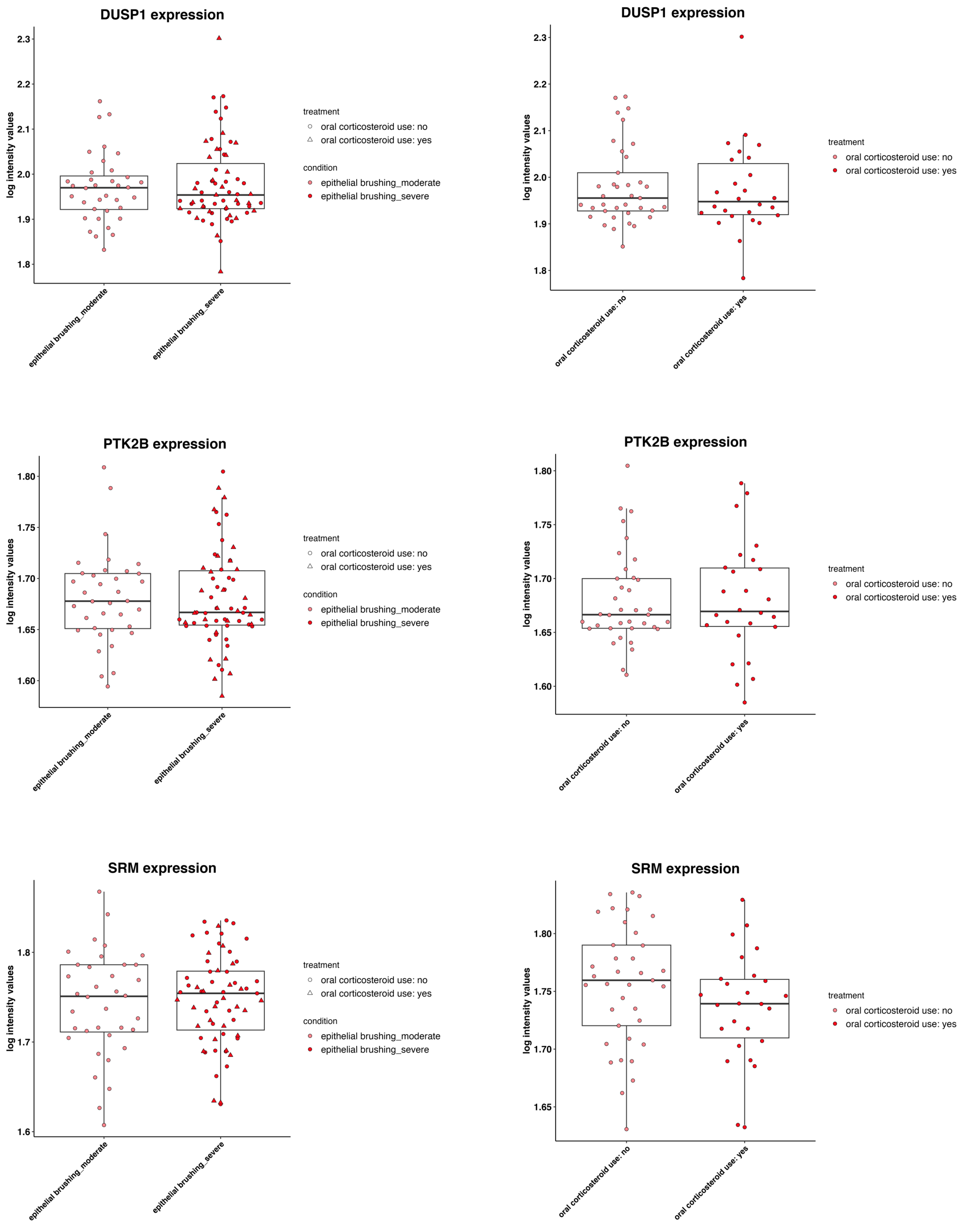

Supplementary Figure 2. mRNA expression in asthma related transcripts in the U-BIOPRED dataset (GSE76227). Microarray data was analyzed using limma. Log intensity values are plotted.

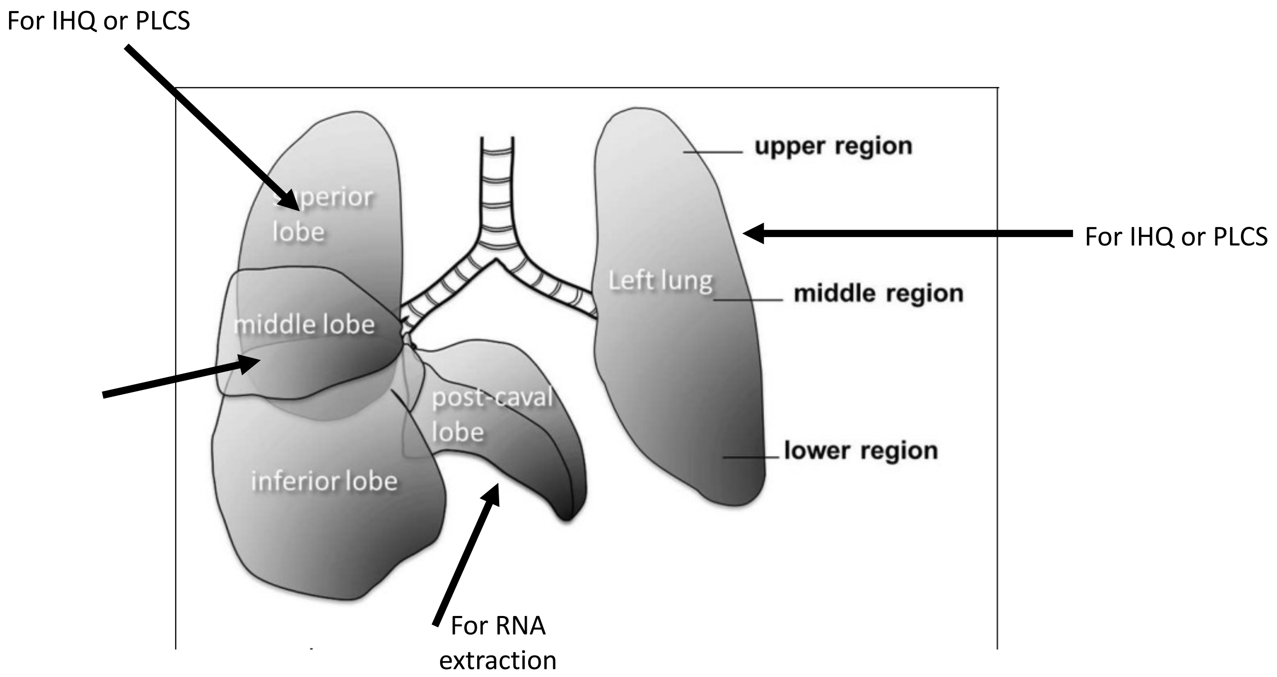

Supplementary Figure 3. Lung regions employed in each experiment. Diagram depicting the regions employed in our mice analyses.

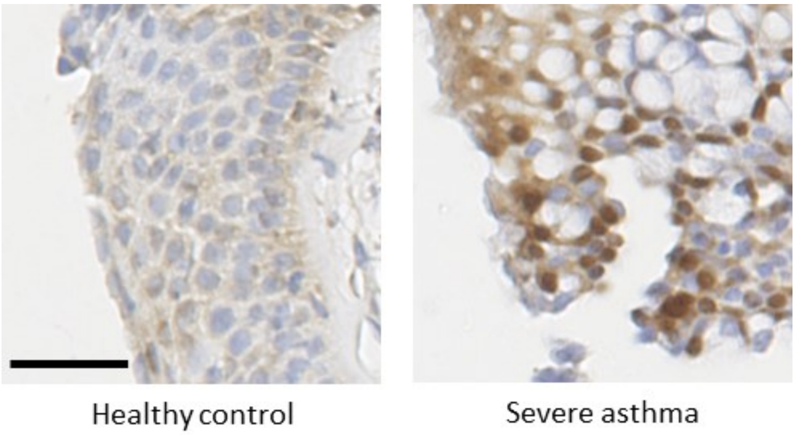

Supplementary Figure 4. Immunohistochemistry staining of ZFP36L2 in bronchial biopsies from healthy controls and patients with severe asthma. Representative images of sections from a patients classified as healthy controls and a patient with severe asthma. Scale bar represents 50 µm.

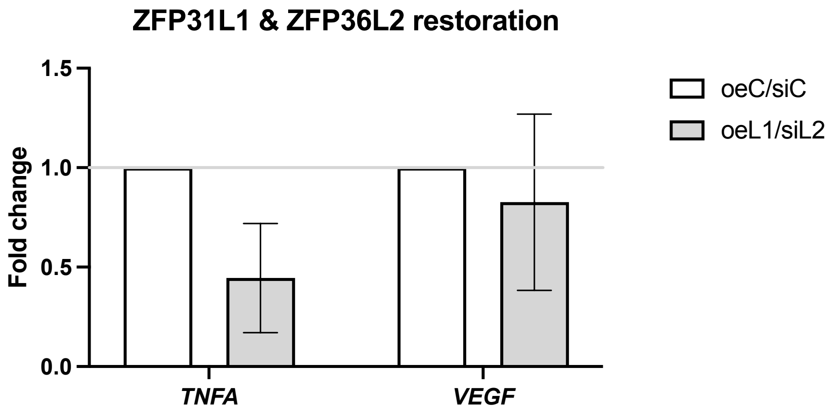

Supplementary Figure 5. Restoration of ZFP36L1/L2 in bronchial epithelial cells from patients with severe asthma does not modulate *TNFA* or *VEGF* mRNA expression.
